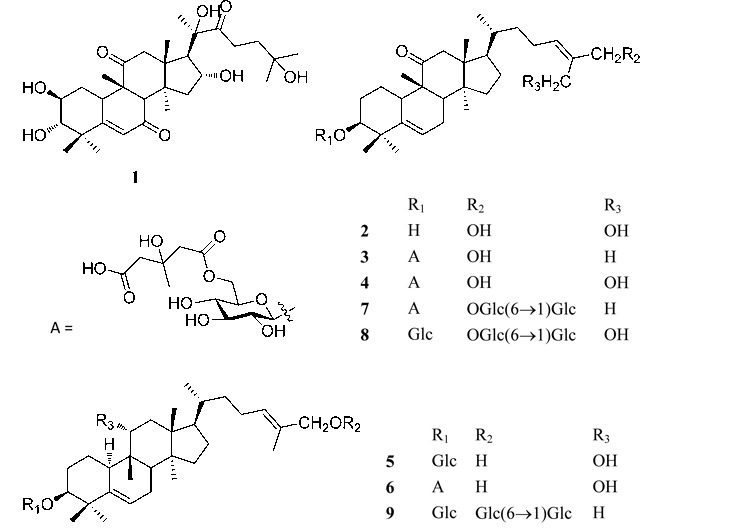# Cucurbitane triterpenoids from *Hemsleya penxianensis*

**DOI:** 10.1007/s13659-011-0044-2

**Published:** 2012-05-24

**Authors:** Jian-Chao Chen, Lin Zhou, Yun-Hua Wang, Ren-Rong Tian, Yun-Xin Yan, Yin Nian, Yun Sun, Yong-Tang Zheng, Ming-Hua Qiu

**Affiliations:** 1 State Key Laboratory of Phytochemistry and Plant Resources in West China, Kunming Institute of Botany, Chinese Academy of Sciences, Kunming, 650201 China; 2 Laboratory of Molecular Immunopharmacology, Key Laboratory of Animal Models and Human Disease Mechanisms, Kunming Institute of Zoology, Chinese Academy of Sciences, Kunming, 650223 China

**Keywords:** cucurbitane triterpenoid, jinfushanencin, jinfushanoside, *Hemsleya penxianensis*

## Abstract

Two new cucurbitacins, jinfushanencins A (**1**) and B (**2**), seven new cucurbitane glycosides, jinfushanosides E-K (**3–9**), along with nine known analogues, were obtained from the tubers of *Hemsleya penxianensis*. Their structures were elucidated on the basis of extensive spectroscopic and chemical methods. Selected isolates were tested their anti-HIV-1 activities, and compound **5** showed weak anti-HIV-1 in C8166 cell (EC_50_ = 5.9 µg/mL) with a selectivity index of 13.5.

## Introduction

The genus *Hemsleya* (Cucurbitaceae), consisting of more than twenty species, are mainly distributed in Yunnan and Sichuan Provinces.[1] The tubers of *Hemsleya* plants are known as famous herbal medicine in China, used in the treatment of bacillary dysentery, bronchitis, and tuberculosis etc.[1] The *Hemsleya* species distributed at Jinfu Mountain was initially recorded as *H. jinfushanensis*, and then as *H. penxianensis* var. *jinfushanensis* by Zhang W. J. and Shen L. T., but was ascribed to *H. penxianensis* by Li D. Z.[2,3] Previously, the phytochemical and bioactive studies of this plant have been reported.[4,5] In continuation of these study, nine new cucurbitane triterpenoids and nine known analogues were obtained. Herein, we describe the isolation and structural elucidation of the new ones.

1

| | R1 | R2 | R3 |
|---|---|---|---|
| 2 | H | OH | OH |
| 3 | A | OH | H |
| 4 | A | OH | OH |
| 7 | A | OGlc(6→1)Glc | H |
| 8 | Glc | OGlc(6→1)Glc | OH |
| 10 | H | OH | H |
| 11 | Glc | OH | H |
| 12 | Glc | OGlc(6→1)Glc | H |
| 13 | Glc | H | OGlc |
| 14 | H | H | OGlc(2→1)Glc |

17

18

| | R1 | R2 | R3 |
|---|---|---|---|
| 5 | Glc | H | OH |
| 6 | A | H | OH |
| 9 | Glc | Glc(6→1)Glc | H |
| 15 | H | Glc(6→1)Glc | OH |
| 16 | Glc | Glc(6→1)Glc | OH |

A =

## Results and Discussion

Jinfushanencin A (**1**) was obtained as colorless powder with the empirical molecular formula of $C_{30}H_{46}O_8$, in agreement with the negative ion HRESIMS (*m/z* 533.3145 $[M - H]^-$, calcd for $C_{30}H_{45}O_8$, 533.3114) and $^{13}C$ NMR spectroscopic data. The IR spectrum revealed absorptions at 3436, 1696, and 1648 $cm^{-1}$, suggestive of hydroxyl and conjugated carbonyl groups. Obvious signals observed in the $^1H$ NMR spectrum were eight methyl singlets at $\delta_H$ 1.24 (3H, s), 1.27 (3H, s), 1.36 (3H × 2, s), 1.40 (3H, s), 1.48 (3H, s), 1.57 (3H, s), and 1.63 (3H, s), an olefinic proton singlet at $\delta_H$ 6.50 (1H, s), as well as two doublets at $\delta_H$ 3.54 (1H, d, $J$ = 9.1 Hz) and 2.92 (1H, d, $J$ = 7.0 Hz). The $^{13}C$ NMR and DEPT spectra revealed 30 carbon signals due to eight methyls, five methylenes, seven

*To whom correspondence should be addressed. E-mail: mhchiu@mail.kib.ac.cn

**Table 1. $^1$H NMR (500 MHz) spectroscopic data of compounds 1 and 2, and the aglycones of 3–9 (in $C_5D_5N$, $\delta$ in ppm, $J$ in Hz)**

| pos. | 1 | 2 | 3 | 4 | 5 | 6 | 7 | 8 | 9 |
|---|---|---|---|---|---|---|---|---|---|
| 1 | 1.69 (1H, m) | 1.63 (1H, m) | 1.62 (1H, m) | 1.63 (1H, m) | 1.97 (1H, m) | 2.10 (1H, m) | 1.65 (1H, m) | 1.64 (1H, m) | 1.89 (1H, m) |
| | 2.60 (1H, m) | 2.06 (1H, m) | 1.89 (1H, m) | 1.90 (1H, m) | 2.86 (1H, m) | 2.95 (1H, m) | 1.95 (1H, m) | 1.92 (1H, m) | 2.13 (1H, m) |
| 2 | 4.22 (1H, m) | 1.87 (2H, m) | 1.89 (1H, m) | 1.93 (1H, m) | 2.08 (1H, m) | 2.13 (1H, m) | 1.91 (1H, m) | 1.90 (1H, m) | 1.92 (1H, m) |
| | | | 2.42 (1H, m) | 2.39 (1H, m) | 2.40 (1H, m) | 2.51 (1H, m) | 2.40 (1H, m) | 2.45 (1H, m) | 2.45 (1H, m) |
| 3 | 3.54 (1H, d, 9.1) | 3.71 (1H, s) | 3.64 (1H, s) | 3.67 (1H, s) | 3.66 (1H, br. s) | 3.69 (1H, br. s) | 3.61 (1H, br. s) | 3.62 (1H, s) | 3.64 (1H, br. s) |
| 6 | 6.50 (1H, s) | 5.68 (1H, d, 5.2) | 5.49 (1H, br. s) | 5.54 (1H, br. s) | 5.50 (1H, br. d, 5.4) | 5.48 (1H, d, 5.5) | 5.49 (1H, br. s) | 5.50 (1H, br. s) | 5.47 (1H, br. s) |
| 7 | | 1.92 (1H, m) | 1.80 (1H, m) | 1.81 (1H, m) | 1.72 (1H, m) | 1.91 (1H, m) | 1.92 (1H, m) | 1.86 (1H, m) | 1.89 (1H, m) |
| | | 2.32 (1H, m) | 2.21 (1H, m) | 2.24 (1H, m) | 2.20 (1H, m) | 2.20 (1H, m) | 2.19 (1H, m) | 2.13 (1H, m) | 2.11 (1H, m) |
| 8 | 2.78 (1H, s) | 1.86 (1H, m) | 1.77 (1H, m) | 1.75 (1H, m) | 1.63 (1H, m) | 1.61 (1H, m) | 1.78 (1H, m) | 1.81 (1H, m) | 1.61 (1H, m) |
| 10 | 3.24 (1H, m) | 2.55 (1H, m) | 2.45 (1H, m) | 2.48 (1H, m) | 2.79 (1H, m) | 2.79 (1H, m) | 2.49 (1H, m) | 2.44 (1H, m) | 2.21 (1H, m) |
| 11 | | | | | 4.15 (1H, m) | 4.13 (1H, m) | | | 1.39 (1H, m) |
| | | | | | | | | | 1.62 (1H, m) |
| 12 | 2.87 (1H, d, 14.9) | 2.49 (1H, d, 14.2) | 2.47 (1H, d, 14.1) | 2.48 (1H, d, 14.1) | 2.14 (2H, m) | 2.12 (2H, m) | 2.51 (1H, d, 14.1) | 2.44 (1H, d, 14.3) | 1.43 (1H, m) |
| | 3.26 (1H, overlap) | 2.97 (1H, d, 14.2) | 2.94 (1H, d, 14.1) | 2.97 (1H, d, 14.1) | | | 2.96 (1H, d, 14.1) | 2.89 (1H, d, 14.3) | 1.64 (1H, m) |
| 15 | 1.84 (1H, d, 17.0) | 1.29 (1H, m) | 1.14 (1H, m) | 1.12 (1H, m) | 1.11 (1H, m) | 1.11 (1H, m) | 1.18 (1H, m) | 1.30 (2H, m) | 1.10 (1H, m) |
| | 2.58 (1H, overlap) | 1.38 (1H, m) | 1.27 (1H, m) | 1.28 (1H, m) | 1.22 (1H, m) | 1.23 (1H, m) | 1.27 (1H, m) | | 1.24 (1H, m) |
| 16 | 4.92 (1H, m) | 1.26 (1H, m) | 1.28 (1H, m) | 1.27 (1H, m) | 1.24 (1H, m) | 1.24 (1H, m) | 1.25 (1H, m) | 1.26 (1H, m) | 1.26 (1H, m) |
| | | 1.90 (1H, m) | 1.95 (1H, m) | 1.93 (1H, m) | 1.90 (1H, m) | 1.98 (1H, m) | 1.91 (1H, m) | 1.90 (1H, m) | 1.95 (1H, m) |
| 17 | 2.92 (1H, d, 7.0) | 1.75 (1H, m) | 1.64 (1H, m) | 1.65 (1H, m) | 1.61 (1H, m) | 1.65 (1H, m) | 1.68 (1H, m) | 1.72 (1H, m) | 1.48 (1H, m) |
| 18 | 1.24 (3H, s) | 0.68 (3H, s) | 0.68 (3H, s) | 0.70 (3H, s) | 0.89 (3H, s) | 0.86 (3H, s) | 0.70 (3H, s) | 0.68 (3H, s) | 0.81 (3H, s) |
| 19 | 1.27 (3H, s) | 1.25 (3H, s) | 1.13 (3H, s) | 1.17 (3H, s) | 1.30 (3H, s) | 1.29 (3H, s) | 1.12 (3H, s) | 1.10 (3H, s) | 0.85 (3H, s) |
| 20 | | 1.38 (1H, m) | 1.40 (1H, m) | 1.38 (1H, m) | 1.38 (1H, m) | 1.38 (1H, m) | 1.39 (1H, m) | 1.26 (1H, m) | 1.40 (1H, m) |
| 21 | 1.57 (3H, s) | 0.84 (3H, d, 6.4) | 0.87 (3H, d, 6.2) | 0.88 (3H, d, 6.4) | 0.98 (3H, d, 6.5) | 0.98 (3H, d, 6.3) | 0.86 (3H, d, 6.4) | 0.81 (3H, d, 6.3) | 0.93 (3H, d, 5.8) |
| 22 | | 1.18 (1H, m) | 1.17 (1H, m) | 1.15 (1H, m) | 1.18 (1H, m) | 1.20 (1H, m) | 1.13 (1H, m) | 1.15 (1H, m) | 1.08 (1H, m) |
| | | 1.58 (1H, m) | 1.58 (1H, m) | 1.47 (1H, m) | 1.58 (1H, m) | 1.57 (1H, m) | 1.49 (1H, m) | 1.48 (1H, m) | 1.46 (1H, m) |
| 23 | 3.26 (1H, m) | 2.16 (1H, m) | 2.02 (1H, m) | 2.15 (1H, m) | 2.02 (1H, m) | 2.09 (1H, m) | 2.11 (1H, m) | 2.15 (2H, m) | 1.99 (1H, m) |
| | 3.47 (1H, m) | 2.30 (1H, m) | 2.20 (1H, m) | 2.30 (1H, m) | 2.12 (1H, m) | 2.22 (1H, m) | 2.28 (1H, m) | | 2.10 (1H, m) |
| 24 | 2.20 (2H, m) | 5.89 (1H, t, 6.8) | 5.71 (1H, t, 7.1) | 5.87 (1H, t, 7.1) | 5.71 (1H, t, 7.2) | 5.74 (1H, t, 7.1) | 5.32 (1H, t, 7.2) | 5.80 (1H, m) | 5.70 (1H, m) |
| 26 | 1.36 (3H, s) | 4.71 (2H, s) | 4.31 (2H, s) | 4.69 (2H, s) | 4.30 (2H, s) | 4.32 (2H, s) | 4.27 (1H, m) | 4.87 (1H, m) | 4.20 (1H, m) |
| | | | | | | | 5.09 (1H, m) | 4.63 (1H, m) | 4.41 (1H, m) |
| 27 | 1.36 (3H, s) | 4.73 (2H, s) | 1.82 (3H, s) | 4.69 (2H, s) | 1.83 (3H, s) | 1.83 (3H, s) | 1.87 (3H, s) | 4.62 (2H, s) | 1.82 (3H, s) |
| 28 | 1.40 (3H, s) | 1.14 (3H, s) | 1.11 (3H, s) | 1.14 (3H, s) | 1.15 (3H, s) | 1.16 (3H, s) | 1.13 (3H, s) | 1.12 (3H, s) | 1.09 (3H, s) |
| 29 | 1.48 (3H, s) | 1.42 (3H, s) | 1.51 (3H, s) | 1.52 (3H, s) | 1.64 (3H, s) | 1.64 (3H, s) | 1.27 (3H, s) | 1.54 (3H, s) | 1.53 (3H, s) |
| 30 | 1.63 (3H, s) | 1.00 (3H, s) | 1.02 (3H, s) | 1.00 (3H, s) | 0.93 (3H, s) | 0.91 (3H, s) | 0.99 (3H, s) | 0.87 (3H, s) | 0.78 (3H, s) |

methines, and ten quaternary carbons (including three carbonyl carbons and one olefinic carbon), which were assigned to a triterpene skeleton. Considering the fact that the tetracyclic triterpenoids isolated thus far from the genus *Hemsleya* are cucurbitane-type compounds, in combination with four characteristic quaternary carbons ($\delta_C$ 44.4 (C-4), 49.2 (C-9), 48.5 (C-13), and 50.7 (C-14)) at high field, compound **1** was tentatively proposed to be a cucurbitacin.[6] Further, comparison of the NMR data of **1** with those of 2$\beta$,3$\beta$,16$\alpha$,20(*R*),25-pentahydroxy-9-methyl-19-norlanost-5-en-7,11,22-trione indicated that these two compounds were structurally almost identical with exception of the configuration of the hydroxyl group at C-3.[7] Detailed comparison of the NMR data of these two compounds disclosed that two proton signals at $\delta_H$ 4.22 (1H, m) and 3.54 (1H, d, $J$ = 9.1 Hz), ascribed to H-2 and H-3 by 2D NMR, in **1**, instead of the signals at $\delta_H$ 4.61 (1H, br. d, $J$ = 10.9 Hz, H-2) and 3.98 (1H, br. s, H-3) in the known compound. All evidences mentioned above suggested that compound **1** had a 2$\beta$,3$\alpha$-diol moiety.

The ROESY correlation observed between $\delta_H$ 1.40 (3H, s, H-28) and $\delta_H$ 3.24 (1H, m, H-10) established an $\alpha$ configuration for C-28, together with the ROESY correlations between $\delta_H$ 4.22 (1H, m, H-2) and H-10, and between $\delta_H$ 1.48 (3H, s, H-29) and $\delta_H$ 3.54 (1H, d, $J$ = 9.1 Hz, H-3), confirmed the 2$\beta$ and 3$\alpha$ substituents. The rings B–D and side chain of **1** were identical to those of 2$\beta$,3$\beta$,16$\alpha$,20(*R*),25-pentahydroxy-9-methyl-19-norlanost-5-en-7,11,22-trione by detailed analysis of the $^{13}$C, $^1$H, HMBC, HSQC, and $^1$H-$^1$H COSY spectra of **1**.

The molecular formula of jinfushanencin B (**2**) was determined to be $C_{30}H_{48}O_4$ by negative ion HRESIMS (found $[M - H]^-$ 471.3487, calcd for 471.3474). From the $^{13}$C NMR data in combination with DEPT spectrum, a ketone carbon, two olefinic methines, two olefinic quaternary carbons, one oxygenated methine, two oxygenated methylenes, four methines, eight methylenes, four quaternary carbons, and six methyls were observed. The NMR data of **2** were very similar with those of carnosiflogenin A (**10**).[8] The difference was that the methyl signal for C-27 in **10** was replaced by an oxygenated methylene ($\delta_C$ 58.5) in **2**, in accordance with the olefinic quaternary carbon resonance at $\delta_C$ 136.1 (C-25) in **10** being shifted downfield to $\delta_C$ 140.9 in **2**. These changes suggested that the methyl at C-27 in **10** was oxygenated to be a hydroxymethyl in **2**. The HMBC correlations from $\delta_H$ 4.73 (s, H-27) to $\delta_C$ 65.4 (t, C-26), 140.9 (s, C-25), and 127.5 (d, C-24), were also supporting evidences for the above deduction.

Jinfushanoside E (**3**) had a molecular formula of $C_{42}H_{66}O_{12}$ determined by HRESIMS, $^{13}$C NMR, and DEPT experiments. The molecular weight of **3** was 144 mass units more than that of delavanoside D (**11**).[9] The $^{13}$C NMR and DEPT data (Tables 1–5) of **3** were very similar with those of **11** exception of six additional resonances observed at $\delta_C$ 171.8 (s, C-1''), 46.4 (t, C-2''), 70.2 (s, C-3''), 46.8 (t, C-4''), 174.9 (s, C-5''), and 28.8 (q, C-6''). In the HMBC spectrum, correlations revealed from $\delta_H$ 3.14 (H-2'') to C-1'', 3'', 4'', 6'' and from $\delta_H$ 3.21 (2H, br. d, $J$ = 14.2 Hz, H-4'') to C-2'', 3'', 5'', 6'' (Figure 1) manifested the additional structural feature in **3** being a hydroxymethyl glutaryl (HMG) group.[10] Additionally, HMBC correlation

**Table 2.** $^{13}$C NMR (125 MHz) spectroscopic data of compounds 1 and 2, and the aglycones of 3–9 (in $C_5D_5N$, $\delta$ in ppm)

| pos. | 1 | 2 | 3 | 4 | 5 | 6 | 7 | 8 | 9 |
|---|---|---|---|---|---|---|---|---|---|
| 1 | 32.8 t | 21.6 t | 22.9 t | 22.1 t | 26.8 t | 26.8 t | 22.2 t | 22.2 t | 22.7 t |
| 2 | 70.3 d | 29.8 t | 28.9 t | 28.0 t | 29.7 t | 29.5 t | 28.3 t | 28.0 t | 29.1 t |
| 3 | 80.4 d | 75.6 d | 88.0 d | 87.0 d | 87.9 d | 87.9 d | 87.1 d | 87.2 d | 87.7 d |
| 4 | 44.4 s | 41.9 s | 42.8 s | 42.0 s | 42.4 s | 42.4 s | 42.4 s | 42.1 s | 41.8 s |
| 5 | 167.9 s | 141.5 s | 142.1 s | 141.4 s | 144.2 s | 144.2 s | 141.3 s | 141.3 s | 143.3 s |
| 6 | 124.5 d | 119.0 d | 119.4 d | 118.6 d | 118.5 d | 118.5 d | 118.6 d | 118.6 d | 118.9 d |
| 7 | 199.8 s | 24.2 t | 24.9 t | 24.2 t | 24.8 t | 24.8 t | 24.2 t | 24.2 t | 24.7 t |
| 8 | 58.9 d | 44.0 d | 44.8 d | 44.1 d | 43.5 d | 43.5 d | 44.0 d | 44.0 d | 44.0 d |
| 9 | 49.2 s | 49.1 s | 49.6 s | 48.8 s | 40.1 s | 40.1 s | 48.8 s | 49.0 s | 35.1 s |
| 10 | 36.5 d | 36.0 d | 35.8 d | 36.1 d | 36.8 d | 36.8 d | 36.2 d | 35.9 d | 38.7 d |
| 11 | 211.1 s | 214.0 s | 214.6 s | 213.6 s | 77.8 d | 77.8 d | 213.9 s | 213.8 s | 32.7 t |
| 12 | 49.2 t | 48.8 t | 49.8 t | 49.2 t | 41.1 t | 41.1 t | 49.0 t | 48.8 t | 28.2 t |
| 13 | 48.5 s | 49.1 s | 49.9 s | 49.1 s | 47.4 s | 47.4 s | 48.8 s | 49.1 s | 46.7 s |
| 14 | 50.7 s | 49.6 s | 50.4 s | 49.7 s | 49.7 s | 49.8 s | 49.1 s | 49.6 s | 49.7 s |
| 15 | 46.7 t | 34.5 t | 35.3 t | 34.6 t | 34.5 t | 34.5 t | 34.6 t | 34.6 t | 34.8 t |
| 16 | 70.2 d | 28.0 t | 28.9 t | 28.0 t | 28.3 t | 28.3 t | 28.3 t | 28.5 t | 30.9 t |
| 17 | 58.7 d | 49.1 d | 50.4 d | 49.7 d | 50.7 d | 50.7 d | 49.7 d | 49.6 d | 50.9 d |
| 18 | 21.1 q | 17.0 q | 17.8 q | 16.9 q | 17.0 q | 17.0 q | 17.0 q | 17.0 q | 15.7 q |
| 19 | 20.4 q | 20.2 q | 21.1 q | 20.3 q | 26.3 q | 26.3 q | 20.4 q | 20.3 q | 28.5 q |
| 20 | 80.0 s | 36.0 d | 36.8 d | 35.9 d | 36.1 d | 36.2 d | 36.0 d | 35.9 d | 36.2 d |
| 21 | 25.4 q | 18.3 q | 19.1 q | 18.7 q | 18.8 q | 18.8 q | 18.6 q | 18.3 q | 19.0 q |
| 22 | 215.9 s | 36.8 t | 37.2 t | 36.8 t | 36.6 t | 36.6 t | 36.0 t | 36.5 t | 36.5 t |
| 23 | 33.8 t | 24.5 t | 24.9 t | 24.5 t | 24.6 t | 24.6 t | 24.9 t | 24.7 t | 25.0 t |
| 24 | 38.5 t | 127.5 d | 125.8 d | 127.8 d | 125.1 d | 125.1 d | 128.9 d | 132.0 d | 129.1 d |
| 25 | 69.0 s | 140.9 s | 137.1 s | 140.8 s | 136.3 s | 136.2 s | 132.3 s | 137.3 s | 132.2 s |
| 26 | 29.9 q | 65.4 t | 68.9 t | 65.6 t | 68.1 t | 68.1 t | 75.3 t | 73.3 t | 75.2 t |
| 27 | 30.1 q | 58.5 t | 14.8 q | 58.7 t | 14.0 q | 14.0 q | 14.3 q | 58.4 t | 14.3 q |
| 28 | 24.8 q | 28.0 q | 29.1 q | 28.3 q | 27.7 q | 27.6 q | 28.3 q | 28.4 q | 28.4 q |
| 29 | 23.1 q | 25.8 q | 26.8 q | 25.9 q | 26.3 q | 26.3 q | 25.9 q | 25.9 q | 26.0 q |
| 30 | 19.8 q | 18.5 q | 19.3 q | 18.3 q | 19.3 q | 19.3 q | 18.3 q | 18.5 q | 18.1 q |

observed from $\delta_H$ 5.03 (d, $J$ = 11.1 Hz, $H_a$-6') and $\delta_H$ 4.71 (m, $H_b$-6') to $\delta_C$ 171.8 (s, C-1'') revealed that the HMG group was linked to C-6' of the sugar unit. After alkaline hydrolysis of **3** with 5 M NaOH solvent, **11** was detected in reaction mixture by TLC. The rings A–D and side chain of **3** were identical to those of **11** by detailed analysis of the $^{13}$C, $^1$H, HMBC, HSQC, and $^1$H-$^1$H COSY spectra of **3.**

**Figure 1.** Key HMBC correlations of compound **3**

Jinfushanoside F (**4**) displayed a quasimolecular ion peak at $m/z$ = 777.4436 [M − H]$^-$ (cald for $C_{42}H_{65}O_{13}$, 777.4425), which was consistent with the molecular formula $C_{42}H_{66}O_{13}$. Comparison of NMR data of **4** (Tables 1–5) with those of jinfushanoside B revealed great similarity.[4] The difference was that the C-6' position of the sugar unit in **4** was ester-linked to a HMG functionality, on the basis of the HMBC correlations from $\delta_H$ 5.04 (d, $J$ = 11.3 Hz, $H_a$-6') and $\delta_H$ 4.72 (m, $H_b$-6') to $\delta_C$ 171.7 (s, C-1''), from $\delta_H$ 3.11 (d, $J$ = 14.0 Hz, $H_a$-2'') and $\delta_H$ 3.08 (d, $J$ = 14.0 Hz, $H_b$-2'') to $\delta_C$ 171.7 (s, C-1''), 70.2 (s, C-3''), 47.0 (t, C-4''), and 28.4 (q, C-6''), and from $\delta_H$ 3.17 (br. d, $J$ = 13.8 Hz, H-4'') to $\delta_C$ 46.7 (t, C-2''), 70.2 (s, C-3''), 175.6 (s, C-5''), and 28.4 (q, C-6''). After alkaline hydrolysis of **4** with 5 M NaOH solvent, jinfushanoside B was detected in reaction mixture by TLC. The rings A–D and side chain of **4** were identical to those of jinfushanoside B by detailed analysis of the $^{13}$C, $^1$H, HMBC, HSQC, and $^1$H-$^1$H COSY spectra of **4**.

The negative ion HRESIMS of jinfushanoside G (**5**) showed a molecular ion peak at 619.4183 [M − H]$^-$, in according with an empirical molecular formula of $C_{36}H_{60}O_8$. The molecular weight of **5** was 162 mass units more than that of 3$\beta$,11$\alpha$,26-trihydroxycucurbita-5,24-diene,[8] which implied an additional hexose unit in **5**. Comparison of the $^{13}$C NMR spectrum of **5** with that of the known compound, an additional glucose signals at $\delta_C$ 107.3 (d), 75.5 (d), 78.7 (d), 71.8 (d), 78.1 (d), and 63.1 (t), as well as the signal of C-3 being shifted downfield by 11.4 ppm to $\delta_C$ 87.9 (d) in **5**, were observed, which indicated an additional glucopyranosyl moiety being linked at C-3 in **5**. This deduction was confirmed by the HMBC correlations from the anomeric proton signal at $\delta_H$ 4.86 (d, $J$ = 7.8 Hz, H-1') to $\delta_C$ 87.9 (d, C-3). The coupling value ($J$ = 7.8 Hz) of anomeric proton suggested the presence of a $\beta$-glucopyranosyl moiety, and the sugar was determined to be a D-glucose by comparison with authentic sample on GC analysis after acid hydrolysis of **5**.

Jinfushanoside H (**6**) was determined to have the molecular formula $C_{42}H_{68}O_{12}$ by negative ion HRESIMS (found [M − H]$^-$ 763.4648, calcd for 763.4632). Comparison of NMR data (Tables 1–5) of **6** with those of **5** displayed great similarity. The difference was that an additional HMG group was ester-linked to the C-6' position of the sugar unit in **6**, which was proved by the HMBC correlations from $\delta_H$ 5.03 (d, $J$ = 11.3 Hz, $H_a$-6') and $\delta_H$ 4.72 (m, $H_b$-6') to $\delta_C$ 171.7 (s, C-1'') and from $\delta_H$ 3.14 (2H, m, H-2'') to $\delta_C$ 70.2 (s, C-3''), 46.8 (t, C-4''), and 28.3 (q, C-6''). After alkaline hydrolysis of **6** with 5 M NaOH solvent, **5** was detected in reaction mixture by TLC. The rings A-D and side chain of **6** were identical to those of **5**

**Table 3. $^{1}$H NMR spectroscopic data of the sugar moieties of compounds 3–9**

| pos. | 3 | 4 | 5 | 6 | 7 | 8 | 9 |
|---|---|---|---|---|---|---|---|
| 3-Glc | | | | | | | |
| 1 | 4.78 (1H, d, 7.6) | 4.79 (1H, d, 7.7) | 4.86 (1H, d, 7.8) | 4.82 (1H, d, 7.8) | 4.84 (1H, d, 7.0) | 4.83 (1H, d, 7.8) | 4.89 (1H, d, 7.7) |
| 2 | 3.94 (1H, m) | 3.95 (1H, m) | 3.93 (1H, m) | 3.94 (1H, m) | 3.92 (1H, m) | 3.92 (1H, m) | 3.93 (1H, m) |
| 3 | 4.13 (1H, m) | 4.14 (1H, m) | 4.19 (1H, m) | 4.16 (1H, m) | 4.24 (1H, m) | 4.22 (1H, m) | 4.20 (1H, m) |
| 4 | 3.98 (1H, m) | 4.00 (1H, m) | 4.18 (1H, m) | 4.14 (1H, m) | 4.18 (1H, m) | 4.20 (1H, m) | 4.18 (1H, m) |
| 5 | 3.96 (1H, m) | 3.97 (1H, m) | 3.91 (1H, m) | 3.95 (1H, m) | 4.10 (1H, m) | 3.93 (1H, m) | 3.90 (1H, m) |
| 6 | 4.71 (1H, m) | 4.72 (1H, m) | 4.37 (1H,m) | 4.72 (1H, m) | 4.69 (1H, m) | 4.37 (1H, m) | 4.34 (1H, m) |
| | 5.03 (1H, br. d, 11.1) | 5.04 (1H, br. d, 11.3) | 4.48 (1H, m) | 5.03 (1H, br. d, 11.3) | 4.98 (1H, m) | 4.51 (1H, m) | 4.50 (1H, m) |
| 26-Glc (inner) | | | | | | | |
| 1 | | | | | 4.84 (1H, d, 7.0) | 4.94 (1H, d, 7.8) | 4.85 (1H, d, 7.7) |
| 2 | | | | | 4.04 (1H, m) | 4.06 (1H, m) | 4.06 (1H, m) |
| 3 | | | | | 4.25 (1H, m) | 4.22 (1H, m) | 4.24 (1H, m) |
| 4 | | | | | 4.19 (1H, m) | 4.23 (1H, m) | 4.19 (1H, m) |
| 5 | | | | | 4.10 (1H, m) | 4.13 (1H, m) | 4.05 (1H, m) |
| 6 | | | | | 4.38 (1H, m) | 4.34 (1H, m) | 4.38 (1H, m) |
| | | | | | 4.95 (1H, m) | 4.85 (1H, m) | 4.95 (1H, m) |
| 26 -Glc (terminal) | | | | | | | |
| 1 | | | | | 5.12 (1H, d, 7.0) | 5.06 (1H, d, 7.7) | 5.14 (1H, d, 7.8) |
| 2 | | | | | 3.95 (1H, m) | 4.07 (1H, m) | 3.98 (1H, m) |
| 3 | | | | | 4.23 (1H, m) | 4.24 (1H, m) | 4.25 (1H, m) |
| 4 | | | | | 4.24 (1H, m) | 4.28 (1H, m) | 4.24 (1H, m) |
| 5 | | | | | 3.94 (1H, m) | 3.96 (1H, m) | 3.95 (1H, m) |
| 6 | | | | | 4.39 (1H, m) | 4.34 (1H, m) | 4.39 (1H, m) |
| | | | | | 4.52 (1H, m) | 4.52 (1H, m) | 4.54 (1H, m) |

by detailed analysis of the $^{13}$C, $^{1}$H, HMBC, HSQC, and $^{1}$H-$^{1}$H COSY spectra of **6.**

Jinfushanoside I (**7**) had the molecular formula $C_{54}H_{86}O_{22}$ based on HRESIMS ([M − H]$^{-}$1085.5546, calcd for 1085.5532) data. The molecular weight of **7** was 144 mass units more than that of carnosifloside III (**12**),[8] which implied an additional HMG group in **7**. Comparison of the $^{13}$C NMR spectrum of **7** with that of the known compound, an additional HMG signals at $\delta_C$ 171.6 (s), 47.3 (t), 70.6 (s), 47.4 (t), 174.8 (s), and 28.3 (q), and the signal of C-6' being shifted downfield to $\delta_C$ 64.6 (t) in **7**, were observed, which indicated that the HMG functionality was ester-linked at C-6' in **7**. This proposal was proved by the observed HMBC correlation from $\delta_H$ 4.98 (m, $H_a$-6') and 4.69 (m, $H_b$-6') to $\delta_C$ 171.6 (s, C-1''). After alkaline hydrolysis of **7** with 5 M NaOH solvent, carnosifloside III (**12**) was detected in reaction mixture by TLC.

Jinfushanoside J (**8**) displayed a quasimolecular ion peak at *m/z* = 957.5050 [M − H]$^{-}$ (cald for $C_{48}H_{77}O_{19}$, 957.5059), in accordance with the molecular formula $C_{48}H_{78}O_{19}$. The molecular weight of **8** was 162 mass units more than that of jinfushanoside D,[4] which implied an additional hexose unit in **8**. In the HMQC-TOCSY spectrum, the anomeric proton at $\delta_H$ 4.83 (1H, d, $J$ = 7.8 Hz, H-1') was correlated with six carbons at $\delta_C$ 107.3 (d), 75.5 (d), 78.7 (d), 71.8 (d), 78.4 (d), and 63.1 (t). This glucose was attached to C-3 based on the HMBC correlation between the anomeric proton at $\delta_H$ 4.83 and the carbon signal at $\delta_C$ 87.2 (d, C-3). During the hydrolysis process of **8**, jinfushanosides B–D were detected in reaction mixture by TLC. The configurations of the anomeric protons of the three glucoses were established to be $\beta$ on the basis of the coupling constants of the anomeric protons, and the sugars were determined to be a D-glucose by comparison with authentic sample on GC analysis after acid hydrolysis of **8**.

Jinfushanoside K (**9**) displayed a quasimolecular ion peak at *m/z* = 927.5336 [M − H]$^{-}$ (cald for $C_{48}H_{77}O_{17}$, 927.5317), consistent with the molecular formula $C_{48}H_{78}O_{17}$. The molecular weight of **9** was 14 mass units less than that of carnosifloside III (**12**).[8] The $^{13}$C NMR data (Tables 2 and 4) of **9** was very similar with that of **12** with the exception of a methylene ($\delta_C$ 32.7) instead of the carbonyl functionality ($\delta_C$ 213.7) at C-11 in **9**, in accordance with the disappearance of the AB coupling system of H-12 in $^{1}$H NMR spectrum. In HMBC spectrum, correlations observed from $\delta_H$ 0.85 (3H, s, H-19) to $\delta_C$ 44.0 (d, C-8), 38.7 (d, C-10), 35.1 (s, C-9), and 32.7 (t), also proved the presence of a methylene at C-11. The configurations of the anomeric protons of the three glucoses were established to be $\beta$ on the basis of the coupling constants of the anomeric protons, and the sugars were determined to be a D-glucose by comparison with authentic sample on GC analysis after acid hydrolysis of **9**. Exception of ring B, the other parts of **9** were identical to those of **12** by detailed analysis of the $^{13}$C, $^{1}$H, HMBC, HSQC, and $^{1}$H-$^{1}$H COSY spectra of **9**. Compound **9** was also the first occurrence of

**Table 4. $^{13}$C NMR spectroscopic data of the sugar moieties of compounds 3–9**

| pos. | 3 | 4 | 5 | 6 | 7 | 8 | 9 |
|---|---|---|---|---|---|---|---|
| 3-Glc | | | | | | | |
| 1 | 107.8 d | 106.6 d | 107.3 d | 107.1 d | 106.9 d | 107.3 d | 107.2 d |
| 2 | 75.8 d | 75.1 d | 75.5 d | 75.3 d | 75.3 d | 75.5 d | 75.6 d |
| 3 | 79.3 d | 78.5 d | 78.7 d | 78.5 d | 78.5 d | 78.7 d | 78.7 d |
| 4 | 72.5 d | 71.8 d | 71.8 d | 71.8 d | 71.7 d | 71.8 d | 71.8 d |
| 5 | 76.1 d | 75.4 d | 78.1 d | 77.8 d | 77.4 d | 78.4 d | 78.4 d |
| 6 | 65.8 t | 64.8 t | 63.1 t | 64.9 t | 64.6 t | 63.1 t | 63.3 t |
| 26 -Glc (inner) | | | | | | | |
| 1 | | | | | 103.4 d | 103.5 d | 103.4 d |
| 2 | | | | | 75.1 d | 75.0 d | 75.1 d |
| 3 | | | | | 78.5 d | 78.5 d | 78.5 d |
| 4 | | | | | 71.7 d | 71.7 d | 71.8 d |
| 5 | | | | | 77.4 d | 77.2 d | 77.3 d |
| 6 | | | | | 70.1 t | 70.1 t | 70.2 t |
| 26-Glc (terminal) | | | | | | | |
| 1 | | | | | 105.5 d | 105. 4 d | 105.5 d |
| 2 | | | | | 75.3 d | 75.3 d | 75.6 d |
| 3 | | | | | 78.5 d | 78.6 d | 78.6 d |
| 4 | | | | | 71.7 d | 71.7 d | 72.1 d |
| 5 | | | | | 78.5 d | 78.2 d | 78.5 d |
| 6 | | | | | 62.8 t | 62.8 t | 62.9 t |

**Table 5. $^1$H and $^{13}$C NMR spectroscopic data of hydroxymethyl glutaryl (HMG) groups in compounds 3, 4, 6, and 7**

| | 3 | | 4 | | 6 | | 7 | |
|---|---|---|---|---|---|---|---|---|
| pos. | $\delta_H$ | $\delta_C$ | $\delta_H$ | $\delta_C$ | $\delta_H$ | $\delta_C$ | $\delta_H$ | $\delta_C$ |
| 1" | | 171.8 s | | 171.7 s | | 171.7 s | | 171.6 s |
| 2" | 3.14 (2H, overlap) | 46.4 t | 3.08 (1H, d, 14.0)<br>3.11 (1H, d, 14.0) | 46.7 t | 3.14 (2H, overlap) | 46.4 t | 2.98 (2H, overlap) | 47.3 t |
| 3" | | 70.2 s | | 70.2 s | | 70.2 s | | 70.6 s |
| 4" | 3.21 (2H, br. d, 14.2) | 46.8 t | 3.17 (2H, br. d, 13.8) | 47.0 t | 3.20 (2H, br. d, 14.3) | 46.8 t | 3.02 (2H, overlap) | 47.4 t |
| 5" | | 174.9 s | | 175.6 s | | 175.0 s | | 174.8 s |
| 6" | 1.77 (3H, s) | 28.8 q | 1.77 (3H, s) | 28.4 q | 1.78 (3H, s) | 28.3 q | 1.80 (3H, s) | 28.3 q |

cucurbitane triterpene without carbonyl or hydroxyl functionalities at C-11 from the genus *Hemsleya*.

Known compounds were identified as carnosiflogenin A (**10**),[8] delavanoside D (**11**),[9] carnosifloside III (**12**),[8] 3$\beta$,27-dihydroxycucurbita-5,24-dien-11-on-3-*O*-$\beta$-D-glucopyranosyl-27-*O*-$\beta$-D-glucopyranoside (**13**),[8] delavanoside A (**14**),[9] 11$\alpha$,26-dihydroxycucurbita-5,24-dien-26-*O*-$\beta$-D-glucopyranosyl(1→6)-$\beta$-D-glucopyranoside (**15**),[8] carnosifloside VI (**16**),[8] hexanorcucurbitacin F (**17**),[12] and scandenogenin A (**18**)[10] by comparison with literature data. Among the known compounds, **13**, **15**, and **18** were firstly isolated as natural products, which had been obtained by acid hydrolysis of carnosifloside IV,[8] carnosifloside VI,[8] and scandenoside R1,[11] respectively.

Compounds **2**, **3**, **5**, **7**, **8**, **9**, **13**, and **15**, were tested for *in vitro* inhibitory effects against HIV replication in C8166 cells. All the compounds were no or weak active (Table 6).

**Table 6. Summary of cytotoxicities and anti-HIV-1 activities of the tested compounds**

| compound | anti-HIV-1 activity, $EC_{50}$ ($\mu$g/mL) | cytotoxicity, $CC_{50}$ ($\mu$g/mL) | selectivity index (SI), $CC_{50}/EC_{50}$ |
|---|---|---|---|
| **2** | 15.7 | 43.7 | 2.8 |
| **3** | 47.0 | 95.6 | 2.0 |
| **5** | 5.9 | 79.5 | 13.5 |
| **7** | 97.1 | > 200 | > 2.1 |
| **8** | 82.6 | > 200 | > 2.4 |
| **9** | 67.0 | > 200 | > 3.0 |
| **13** | 42.9 | 111.00 | 2.6 |
| **15** | 78.2 | 108.4 | 1.4 |
| AZT | 0.003 | > 200 | > 50,000 |

## Experimental Section

**General Experimental Procedures.** Melting points were obtained on an XRC-1 apparatus and are uncorrected. Optical rotations were carried out on a Perkin-Elmer model 241 polarimeter. UV spectrum was measured in a UV 210A spectrometer. IR spectra were measured in a Bio-Rad FTS-135 spectrometer with KBr pellets. MS were recorded on a Finnigan MAT 90 instrument. 1D and 2D NMR spectra were measured on a Bruker DRX-500 instrument with TMS as internal standard. Column chromatography was performed either on silica gel (200–300 mesh; Qingdao Marine Chemical Inc., Qingdao, China), or Lichroprep RP-18 gel (40–63 $\mu$m; Merck, Darmstadt, Germany). Fractions were monitored by TLC, and spots were visualized by heating silica gel plates sprayed with 15% $H_2SO_4$ in $H_2O$.

**Plant Material.** The tubers of *H. penxianensis* were collected in Jinfu Mountain, Congqing City, China, in 2000. A voucher specimen (No. KIB 2000-9-4) has been deposited at the State Key Laboratory of Phytochemistry and Plant Resources in West China, Kunming Institute of Botany, Chinese Academy Sciences. The plant material was identified by Prof. Wen-Jin Zhan, Penzhou Institute for Pharmaceutical Control, Sichuan.

**Extraction and Isolation.** Air-dried and powdered tubers (2.0 kg) of *H. penxianensis* were extracted with 95% ethanol under reflux (3 × 8 L), and filtered. After concentration of the combined filtrate under vacuum, 411 g residue was gotten. The residue (322 g) was absorbed on silica gel and chromatographed on silica gel column, eluting with a gradient system of $CHCl_3$, $CHCl_3$-$Me_2CO$ (15:1, 9:1), and $CHCl_3$-EtOH (9:1, 7:3) to give six fractions (Frs. 1–6). Fraction 3 (2.83 g) was fractioned on silica gel, developing with a gradient system of $CHCl_3$-MeOH (20:1, 15:1, 12:1) to furnish three parts (Fr. 3.1–3.3). Fr. 3.3 (1.1 g) was rechromatographed on silica gel, eluting with $CHCl_3$-$CH_3OH$ (15:1, 12:1), then purified on LH-20 using MeOH as eluent to give **1** (12 mg), **2** (57 mg), **10** (17 mg), **17** (32 mg), and **18** (15 mg). Fr. 4 (12.8 g) was subjected to CC (20 g) to generate four fractions (Frs. 4.1–4.4). Fr. 4.3 (8.43 g) was repeatedly subjected to silica gel column eluting with $CHCl_3$-$CH_3OH$ (10:1) and $RP_{18}$ silica gel developing with aqueous MeOH (60% → 70%), to generate **3** (174 mg), **4** (19 mg), **5** (47 mg), **6** (23 mg), and **11** (86 mg). Compounds **13** (42 mg), **14** (31 mg), and **15** (19 mg) were isolated from Fr.4.4 (1.5 g) by subjected to column chromatography over silica gel ($CHCl_3$-MeOH, from 6:1 to 9:1) and RP-18 (aqueous MeOH, from 55% to 60%), then Sephadex LH-20 (MeOH). Fr. 5 (131 g) was rechromatographed on silica gel developing with a gradient system of $CHCl_3$-MeOH-$H_2O$ (8:1:0.1, 8:2:02, 7:3:1, 7:3:0.5) to yield Fr. 5.1–Fr. 5.5. Fr.5.3 (9 g) was further rechromatographed over $RP_{18}$ silica gel developing with aqueous MeOH (40% → 50%) to afford compounds **9** (39 mg), **12** (451 mg), and **16** (320 mg). Compounds **7** (150 mg) and **8** (164 mg) were isolated from Fr.5.4 (4 g) by subjected to column chromatography over RP-18 silica gel, eluting with a gradient aqueous MeOH system from 40% to 45%.

**Jinfushanencin A (1):** colorless powder; mp 217–219 °C; $[\alpha]_D^{20}$ + 22.0 (*c* 0.05, MeOH); IR (KBr) $\nu_{max}$: 3436, 2973, 1696, 1648, 1302 cm$^{-1}$; $^1$H and $^{13}$C NMR data, see Tables 1 and 2; negative FABMS: *m/z* 533 [M – H]$^-$; negative HRESIMS: *m/z* 533.3145 [M – H]$^-$ (calcd for $C_{30}H_{45}O_8$, 533.3114).

**Jinfushanencin B (2):** colorless powder; mp 149–151 °C; $[\alpha]_D^{20}$ + 15.8 (*c* 0.12, MeOH); IR (KBr) $\nu_{max}$: 3372, 2952, 2929, 2874, 1693, 1464, 1382, 1028, 1006, 979 cm$^{-1}$; $^1$H and $^{13}$C NMR data, see Tables 1 and 2; negative FABMS: *m/z* 471 [M

– H]$^-$; negative HRESIMS: *m/z* 471.3487 [M – H]$^-$ (calcd for $C_{30}H_{47}O_4$, 471.3474).

**Jinfushanoside E (3):** white powder; $[\alpha]_D^{20}$ + 8.6 (*c* 0.36, MeOH); IR (KBr) $\nu_{max}$: 3425, 2962, 2878, 1728, 1633, 1383, 1206, 1078, 978 cm$^{-1}$; $^1$H and $^{13}$C NMR data, see Tables 1–5; negative FABMS: *m/z* 761 [M – H]$^-$; negative HRESIMS: *m/z* 761.4491 [M – H]$^-$ (cald for $C_{42}H_{65}O_{12}$, 761.4476).

**Jinfushanoside F (4):** white powder; $[\alpha]_D^{20}$ + 9.2 (*c* 0.24, MeOH); IR (KBr) $\nu_{max}$: 3424, 3369, 2962, 2877, 1726, 1692, 1383, 1207, 1077, 1038 cm$^{-1}$; $^1$H and $^{13}$C NMR data, see Tables 1–5; negative FABMS: *m/z* 777 [M – H]$^-$; negative HRESIMS: *m/z* 777.4436 [M – H]$^-$ (cald for $C_{42}H_{65}O_{13}$, 777.4425).

**Jinfushanoside G (5):** white powder; $[\alpha]_D^{20}$ + 12.0 (*c* 0.09, MeOH); IR (KBr) $\nu_{max}$: 3409, 3368, 2942, 2928, 1638, 1464, 1381, 1075, 1030 cm$^{-1}$; $^1$H and $^{13}$C NMR data, see Tables 1–4; negative FABMS: *m/z* 619 [M – H]$^-$; negative HRESIMS: *m/z* 619.4183 [M – H]$^-$ (cald for $C_{36}H_{59}O_8$, 619.4209).

**Jinfushanoside H (6):** white powder; $[\alpha]_D^{20}$ + 8.1 (*c* 0.09, MeOH); IR (KBr) $\nu_{max}$: 3431, 2928, 2874, 1722, 1453, 1076, 981 cm$^{-1}$; $^1$H and $^{13}$C NMR data, see Tables 1–5; negative FABMS: *m/z* 763 [M – H]$^-$; negative HRESIMS: *m/z* 763.4648 [M – H]$^-$ (cald for $C_{42}H_{67}O_{12}$,763.4632).

**Jinfushanoside I (7):** white powder; $[\alpha]_D^{20}$ + 5.9 (*c* 0.09, MeOH); IR (KBr) $\nu_{max}$: 3431, 2945, 2875, 1729, 1579, 1458, 1076, 1021 cm$^{-1}$; $^1$H and $^{13}$C NMR data, see Tables 1–5; negative FABMS: *m/z* 1085 [M – H]$^-$; negative HRESIMS: *m/z* 1085.5546 [M – H]$^-$ (cald for $C_{54}H_{85}O_{22}$,1085.5532).

**Jinfushanoside J (8):** white powder; $[\alpha]_D^{20}$ + 12.6 (*c* 0.11, MeOH); IR (KBr) $\nu_{max}$: 3452, 3283, 2926, 2877, 1690, 1467, 1075, 1020 cm$^{-1}$; $^1$H and $^{13}$C NMR data, see Tables 1–4; negative FABMS: *m/z* 957 [M – H]$^-$; negative HRESIMS: *m/z* 957.5050 [M – H]$^-$ (cald for $C_{48}H_{77}O_{19}$, 957.5059).

**Jinfushanoside K (9):** white powder; $[\alpha]_D^{20}$ – 5.3 (*c* 0.17, MeOH); IR (KBr) $\nu_{max}$: 3409, 2935, 2872, 1640, 1460, 1379, 1075, 1038 cm$^{-1}$; $^1$H and $^{13}$C NMR data, see Tables 1–4; negative FABMS: *m/z* 927 [M – H]$^-$; negative HRESIMS: *m/z* 927.5336 [M – H]$^-$ (cald for $C_{48}H_{77}O_{17}$, 927.5317).

**Acid Hydrolysis of 5, 8, and 9:** Each of the compounds **5**, **8**, and **9** (2 mg) dissolved in 2 mL HCl-$CH_3OH$ (1 mol L$^{-1}$ HCl-$CH_3OH$, 1:1, v/v) was placed in water bath at 90 °C for 6 hrs. After the hydrolysis was finished, the water-soluble part was evaporated to dryness. The dry sugar residue and authentic sample of D-(+)-glucose (Sigma Company, G5250) were respectively diluted in 1 ml pyridine without water and treated with 0.4 mL hexamethyl disilazane and 0.2 mL trimethylchlorosilane (TMCS, Fluka) at 0 °C. After that, the upper layer of the reaction mixture was analyzed by GC. (GC condition: AC-5 capillary column (30 m × Ø0.25 mm); column temperature: 180-260 °C; column head pressure: 12 Pa; carrier gas: $N_2$). GC of all samples showed same retention time, $R_t$ (m): 11.521.

**Partial Alkaline Hydrolysis of 3, 4, 6, and 7:** A solution of the triterpene glycoside (each 0.5 mg dissolved in 1 ml MeOH) was added 0.5 % aq. NaOH (20 mL). After 2 h shaking at room temperature, the reaction mixture was acidified to PH 5 (5 M HCl) and was concentrated to dry. The resulting residue was dissolved in MeOH, and then was checked by using TLC. As a result, delavanoside D (**11**), jinfushanoside B, jinfushanoside G (**5**), and carnosifloside III (**12**), were detected in the reaction mixture of **3**, **4**, **6**, and **7**, by TLC, respectively.

**Anti-HIV-1 and Cytotoxicity Assay.** The anti-HIV-1 activity was evaluated by the inhibition assay for the cytopathic effects of HIV-1 ($EC_{50}$), and cytotoxicity assay against C8166 cells ($IC_{50}$) was assessed using the MTT method as described in the literature.[13]

**Acknowledgments**

This work was financially supported by the Natural Science Foundation of Yunnan Province (2008CD158), the Western Doctoral Foundation of Chinese Academy of Sciences (J.C. Chen), the Foundation of State Key Laboratory of Phytochemistry and Plant Resources in West China, Kunming Institute of Botany, Chinese Academy of Sciences (P2008ZZ23, P2010ZZ14), and the Cooperative Project of Guangdong Province and CAS (2009B091300135).

**Open Access** This article is distributed under the terms of the Creative Commons Attribution License which permits any use, distribution, and reproduction in any medium, provided the original author(s) and source are credited.
